# Late blight resistance genes in potato breeding

**DOI:** 10.1007/s00425-022-03910-6

**Published:** 2022-05-16

**Authors:** Paulina Paluchowska, Jadwiga Śliwka, Zhimin Yin

**Affiliations:** Plant Breeding and Acclimatization Institute-National Research Institute, Platanowa 19, 05-831 Młochów, Poland

**Keywords:** Cultivar, Effector, Genetic engineering, *Phytophthora infestans*, *Solanum tuberosum*, Wild crop relatives

## Abstract

**Main conclusion:**

Using late blight resistance genes targeting conservative effectors of *Phytophthora infestans* and the constructing gene pyramids may lead to durable, broad-spectrum resistance, which could be accelerated through genetic engineering.

**Abstract:**

Potato (*Solanum tuberosum* L.) is one of the most important food crops worldwide. In 2020, potato production was estimated to be more than 359 million tons according to the Food and Agriculture Organization (FAO). Potato is affected by many pathogens, among which *Phytophthora infestans*, causing late blight, is of the most economic importance. Crop protection against late blight requires intensive use of fungicides, which has an impact on the environment and humans. Therefore, new potato cultivars have been bred using resistance genes against *P. infestans* (*Rpi* genes) that originate from wild relatives of potato. Such programmes were initiated 100 years ago, but the process is complex and long. The development of genetic engineering techniques has enabled the direct transfer of resistance genes from potato wild species to cultivars and easier pyramiding of multiple *Rpi* genes, which potentially increases the durability and spectrum of potato resistance to rapidly evolving *P. infestans* strains. In this review, we summarize the current knowledge concerning *Rpi* genes. We also discuss the use of *Rpi* genes in breeding as well as their detection in existing potato cultivars. Last, we review new sources of *Rpi* genes and new methods used to identify them and discuss interactions between *P. infestans* and host.

## Introduction

Potato (*Solanum tuberosum* L*.*) plants are cultivated worldwide; the largest areas can be found in Asia and Europe and potato production is systematically increasing in Africa (Haverkort and Struik 2015). Late blight is the most economically important potato disease. Costs associated with crop loss and chemical control of late blight were estimated to be more than € 9 billion per year (Haverkort et al. 2016). Late blight is caused by *Phytophthora infestans* (Mont.) de Bary*,* an oomycete within the kingdom Stramenopiles, which also infects tomato (*Solanum lycopersicum* L.) plants. This pathogen can infect stems, berries, leaves and tubers, which leads to complete crop loss. In the nineteenth century, *P. infestans* caused severe destruction of potato crops in Europe, especially in Ireland, where potatoes were the staple food (Kamoun et al. 2015). Intensive research on potato late blight has led to the discovery of dominant resistance genes against *P. infestans* (*Rpi* genes) in potato wild species. Research was initiated to introduce the *Rpi* genes from *Solanum demissum* into potato cultivars (Black et al. 1953; Malcolmson and Black 1966). Potato cultivars carrying resistance genes derived from *S. demissum*, including Pentland Ace (*R3*), Pentland Dell (*R1, R2* and *R3*) and Epoka (*R4*), have been registered and cultivated on a large scale in Europe (Malcolmson 1969; Rudkiewicz 1985). However, *Rpi* genes introduced from *S. demissum* were quickly overcome by new virulent *P. infestans* strains (Jo et al. 2014). *Rpi* gene introgression from wild relatives of the potato into commercial cultivars through crossing is time-consuming, especially in the case of species separated from potato with crossing barriers such as different endosperm balance numbers (EBNs). For example, the introgression of a single *Rpi* gene (*Rpi-blb2*) from the wild species *Solanum bulbocastanum* to potato cultivars Bionica and Toluca necessitated more than 45 years (Haverkort et al. 2016). Compared with conventional breeding, genetic engineering techniques such as cisgenesis facilitate a faster introduction of *Rpi* genes into commercial cultivars (Ghislain et al. 2019). However, to avoid a rapid overcoming of resistance in newly engineered cultivars, introducing not one but several *Rpi* genes at a time has been proposed (Haverkort et al. 2016).

In this review, we summarize the knowledge concerning *Rpi* genes. We discuss the use of *Rpi* genes in traditional and genetic modification-based breeding as well as their detection in existing potato cultivars. We present new sources of *Rpi* genes and new methods used to identify them and discuss the interactions between *P. infestans* and the host.

## Sources of *Rpi* genes

Hawkes’s taxonomy originally distinguished 232 potato wild species (Hawkes 1990). However, recent morphological and molecular studies have reduced the number of potato wild species to 107 (Spooner et al. 2016). These wild species grow in America from the southwestern United States to central Argentina and Chile (Hijmans and Spooner 2001). The highest number of species (93) occurs in Peru, 43 of which can be described as rare. Another country where species richness is particularly high is Mexico, which has 36 potato wild species (Hijmans and Spooner 2001).

Wild relatives of the potato are unique sources of genetic variation. They are characterized as being highly resistant to various diseases, including late blight, and they have been used in breeding programmes for more than 100 years (Machida-Hirano 2015). To date, more than 70 *Rpi* genes have been identified and mapped in 32 *Solanum* species (Table 1). Most of the *Rpi* genes have been derived from tuber-bearing species (25): Mexican (9 species), Bolivian (6), Peruvian (4), Argentine (3), Paraguayan (1), USA (1) and one species found generally in the Andes. Novel *Rpi* genes were found also in *S. tuberosum* subspecies *andigena* and in Hungarian cultivar Sárpo Mira. Six *Rpi* genes were identified in four non-tuber-bearing species and five from the tomato wild species *S. pimpinellifolium*. Single resistance genes were identified in 15 potato wild species. Frequently, multiple functional *Rpi* genes have been found within a single species, e.g., *S. demissum* (14 *Rpi* genes), *S. bulbocastanum* (5), *S. berthaultii* (5), *S. stoloniferum* (4), *S. edinense* (4), *S. venturii* (4), *S. hjertingii* (3), *S. chacoense* (3), *S. huancabambense* (2), *S. pinnatisectum* (2) *S. schenckii* (2) and *S. tarijense* (2). The *Rpi* genes were mapped in clusters onto potato chromosomes I, IV, V, VI, VII, VIII, IX, X, and XI. For example, on chromosome IV, a total of 13 *Rpi* genes from seven potato wild species were found. Several *Rpi* genes have not yet been mapped, including the following: *Rpi-pta2* from *S. stoloniferum*; *R4*^*BI*^ and *R4*^*MA*^ from *S. demissum*; *Rpi-ber1.2, Rpi-ber1.3,* and *Rpi-ber1.4* from *S. berthaultii*; *Rpi-tar1.3* from *S. tarijense, Rpi-nrs1* from *S. neorossii* and putative novel *Rpi* genes from *S. jamesii* and *S. tuberosum* subsp. *andigena* (Table 1).

**Table 1:** Resistance genes against *Phytophthora infestans* (*Rpi* genes) in *Solanum* species

*Rpi* gene	Species	Chromosome	Origin	References
Wild potato relatives				
*Rpi-avl1*	*S. avilesii*	XI	Bolivia	Verzaux (2010)
*Rpi-ber1; Rpi-ber2*	*S. berthaultii*	X		Park et al. (2009)
*Rpi-ber1.2; Rpi-ber1.3; Rpi-ber1.4*		NA		Monino-Lopez et al. (2021)
*Rpi-blb1 (RB)*^a^	*S. bulbocastanum*	VIII	Mexico	Naess et al. (2000)
*Rpi-blb2*^a^		VI		Van der Vossen et al. (2005)
*Rpi-blb3*^a^*; Rpi-abpt*		IV		Park et al. (2005a); Lokossou et al. (2009)
*Rpi-bt1*		VII		Oosumi et al. (2009)
*Rpi-chc1.1; Rpi-chc1.2; Rpi-chc2*	*S. chacoense*	X	Paraguay	Monino-Lopez et al. (2021); Haverkort et al. (2016)
*Rpi-cap1*	*S. capsicibaccatum*	XI	Bolivia	Verzaux et al. (2012)
*Rpi-qum1*	*S. circaeifolium* ssp. *quimense*	XI		
*R1*	*S. demissum*	V	Mexico	Ballvora et al. (2002)
*R2; Rpi-demf1*		IV		Lokossou et al. (2009); Danan et al. (2011)
*R3a; R3b*		XI		El-Kharbotly et al. (1996); Huang et al. (2004)
*R4*^*aI*^; *R4*^*MA*^		NA		Van Poppel (2010)
*R5*		XI		Huang (2005)
*R6; R7*		XI		El-Kharbotly et al. (1996); Huang (2005)
*R8* (*Rpi-Smira2*)^a^*; R9a* (*Rpi-edn2*)^a^		IX		Jo et al. (2011); Vossen et al. 2016; Jo et al. 2015; Keijzer et al. (2021)
*R10; R11*		XI		Bradshaw et al. (2006)
*Rpi-edn1.1; Rpi-edn1.2*	*S. edinense*	IV		Champouret (2010)
*Rpi-edn2 (R9a)*^a^		IX		Verzaux (2010); Keijzer et al. (2021)
*Rpi-edn3*		XI		Verzaux (2010);
*Rpi-hjt1.1; Rpi-hjt1.2; Rpi-hjt1.3*	*S. hjertingii*	IV		Champouret (2010)
*Rpi-hcb1.1; Rpi-hcb1.2*	*S. huancabambense*	IX	Peru	Aguilera-Galvez et al. (2020)
Novel *Rpi* gene(s)	*S. jamesii*	NA	USA	Zheng et al. (2020)
*Rpi-mch1*	*S. michoacanum*	VII	Mexico	Śliwka et al. (2012b)
*Rpi-mcd1*	*S. microdontum*	IV	Argentina	Sandbrink et al. (2000)
*Rpi-mcq1* (*Rpi-moc1*)	*S. mochiquense*	IX	Peru	Smilde et al. (2005)
*Rpi-nrs1*	*S. neorossii*	IX	Argentina	Jones et al. (2009)
*Rpi-pcs*	*S. paucissectum*	XI	Peru	Villamon et al. (2005)
*Rpi-phu1* (*Rpi-vnt1.1*)^a^	*S. phureja*	IX	Andes	Śliwka et al. (2006); Foster et al. (2009)
*Rpi1; Rpi2*	*S. pinnatisectum*	VII	Mexico	Kuhl et al. (2001); Yang et al. (2017)
*Rpi-pur1*	*S. piurae*	XI	Peru	Rietman (2011)
*Rpi-rzc1*^a^	*S. ruiz-ceballosii* (*S. brevicaule*)	X	Bolivia	Śliwka et al. (2012a)
*Rpi-snk1.1; Rpi-snk1.2*	*S. schenckii*	IV	Mexico	Champouret (2010)
*Rpi-sto1*^a^*; Rpi-pta1*	*S. stoloniferum*	VIII		Vleeshouwers et al. (2008); Wang et al. (2008)
*Rpi-sto2*		XI		Champouret (2010)
*Rpi-pta2*		NA		Vleeshouwers et al. (2008); Wang et al. (2008)
*Rpi-tar1*	*S. tarijense*	X	Bolivia	Haverkort et al. (2016)
*Rpi-tar1.3*		NA		Monino-Lopez et al. (2021)
*Rpi-Smira1*	*S. tuberosum* cv. Sárpo Mira	XI	Hungary	Rietman et al. (2012); Tomczyńska et al. 2014; Vossen et al. 2016
*Rpi-Smira2 (R8)*^a^		IX		
Novel *Rpi* gene(s)	*S. tuberosum* subsp. *andigena*	NA	South America	Duan et al. (2020)
*Rpi-vnt1.1 (Rpi-phu1); Rpi-vnt1.2; Rpi-vnt1.3*^a^	*S. venturii*	IX	Argentina	Foster et al. (2009); Śliwka et al. (2006)
*Rpi-vnt2*		XI		Rietman (2011)
*Rpi-ver1*	*S. verrucosum*	IX	Mexico	Chen et al. (2018)
Non-tuber-bearing *Solanum* species				
*Rpi-amr3*^a^	*S. americanum*	IV	Mexico	Witek et al. (2016)
*Rpi-amr1-2273*^a^		XI	NA	Witek et al. (2021)
*Rpi-amr1-3409*^a^	*S. nigrescens*	I	NA	
*Rpi-dlc1*	*S. dulcamara*	IX		Golas et al. (2010) Golas et al. (2013)
*Rpi-dlc2*		X		
*Rpi-crp1*	*S. caripense*	IX	Andes	Nakitandwe (2007)
Wild tomato relatives				
*Ph-1*	*S. pimpinellifolium*	VII	Peru; Ecuador	Bonde and Murphy (1952)
*Ph-2*		X		Gallegly and Marvel (1955)
*Ph-3*		IX		Chunwongse et al. (2002)
*Ph-5.1*		I		Merk and Foolad (2012)
*Ph-5.2*		X		

NA, not available

^a^*Rpi* genes described as providing durable resistance against late blight in literature

Recently, using advanced techniques, new *Rpi* genes have been identified. Through genetic linkage analysis and collinearity analysis, a new dominant resistance gene, *Rpi2*, from the Mexican diploid wild species *S. pinnatisectum* was mapped onto potato chromosome VII (Yang et al. 2017). The *Rpi2* locus is different from the previously reported resistance locus *Rpi1*, which is on the same chromosome. *Rpi2* provides broad-spectrum resistance against various *P. infestans* isolates, including those that overcome resistance conferred by *R9*. Resistance gene enrichment sequencing (RenSeq) was used to finely map onto chromosome X, the *Rpi-rzc1* gene from *S. ruiz-ceballosii*, which confers high and broad-spectrum resistance to 500 diverse Polish *P. infestans* isolates, (Jupe et al. 2013; Brylińska et al. 2015). Two complementary enrichment strategies that target resistance genes (RenSeq) and single/low-copy number genes (GenSeq, generic-mapping enrichment sequencing) independently positioned the broad-spectrum resistance gene *Rpi-ver1* from the Mexican wild species *S. verrucosum* on potato chromosome IX (Chen et al. 2018). Diploid wild potato, *S. jamesii* (JAMI-4) is completely resistant to the super virulent *P. infestans* isolate 2013–18-306, which can overcome the resistance conferred by the genes *R1, R2, R3a, R3b, R4, R5, R6, R7, R8, R9, R10,* and *R11* (Zheng et al. 2020). Diagnostic RenSeq (dRenSeq) analysis demonstrated JAM1-4 harbors *R3a*. However, transgenic Désirée plants containing *R3a* are susceptible to the isolate 2013–18-306. The authors speculated that resistance in JAM1-4 was provided by uncharacterized novel resistance gene(s). *Rpi-amr3* from *S. americanum* was identified and cloned via RenSeq and single-molecule real-time (SMRT) sequencing (SMRT RenSeq) (Witek et al. 2016). Bulked segregant analysis coupled with RenSeq mapped *Rpi-amr3* on chromosome IV of the potato reference genome of a doubled monoploid clone of *S. tuberosum* group Phureja DM1–3 516 R44 (DM). Transgenic diploid potato carrying *Rpi-amr3* showed resistance against three *P. infestans* isolates. Another new *Rpi* gene from *S. americanum Rpi-amr1*, was positionally cloned and mapped onto the short arm of chromosome XI (Witek et al. 2021). Using association genomics and long-read RenSeq, the authors identified three allele-specific proteins, which showed 100% identity to Rpi-amr1 protein, from two *S. americanum* assessions and one *S. nodiflorum* assession. Eight additional Rpi-amr1 allele-specific proteins, sharing 90% homology to Rpi-amr1 proteins, were identified from six accessions of *S. americanum* and two accessions of *S. nigrescens* and they all conferred late blight resistance to *P. infestans* isolate 88069 in transient assays. One homologue, *Rpi-amr1-3409* from *S. nigrescens*, was mapped onto chromosome I based on the potato DM reference genome, suggesting that a fragment of DNA from the end of the short arm of chromosome XI in other resistant accessions was translocated to the end of the long arm of chromosome I in *S. nigrescens*. Moreover, the authors identified *Rpi-amr1* homologues in hexaploid *S. nigrum* accessions, providing resistance to the *P. infestans* isolate 88069. Previous studies indicated that *S. nigrum* is a non-host to *P. infestans* and *S. americanum* may be the diploid ancestor of hexaploid *S. nigrum* (Colon et al. 1992; Poczai and Hyvönen 2010). The *Rpi-amr1* homologues, which confer late blight resistance in *S. nigrum*, were most likely inherited from *S. americanum* (Witek et al. 2021). *Rpi-amr1* confers broad-spectrum late blight resistance in cultivated potato. Stably transformed transgenic potato cultivar Maris Piper plants carrying *Rpi-amr1*, resist 19 *P. infestans* isolates tested, including those overcoming *Rpi-vnt1*, *Rpi-blb1* and *Rpi-blb2*. In potato wild species *S. chacoense,* two resistance genes, *Rpi-chc1.1* and *Rpi-chc1.2* have been identified (Monino-Lopez et al. 2021). An allele-mining strategy allowed the identification of *Rpi-chc1.1* orthologue in *S. chacoense*, *S. berthaultii* and *S. tarijense* accessions resistant to late blight. For many years, researchers have continued to search for new *Rpi* genes among wild potato. The largest collections of potato germplasm are available in International Potato Center (CIP) in Peru, the USDA Potato Genebank in Wisconsin, USA, and IPK Gatersleben Genebank in Germany (Karki et al. 2021b). An analysis of resistance to *P. infestans* carried out over a period of more than 20 years has shown that among 34 potato wild relatives there are accessions characterized by a high level of resistance, but the genes underlying this resistance are still unknown (Pérez et al. 1999; Zoteyeva et al. 2012; Khiutti et al. 2015; Bachmann-Pfabe et al. 2019; Zoteyeva 2020; Karki et al. 2021b). A list of such potato wild relatives is shown in Table 2. These species are native to Mexico, Argentina, Bolivia, Peru, Ecuador and Chile. Research using aggressive *P. infestans* isolates, showed that these species can contribute to development of new durable resistant cultivars. It is worth noting that species having 2EBN and 4EBN (Table 2) can cross with cultivated potato and can be used in potato breeding programs (Karki et al. 2021b). On the other hand, species with 1EBN cannot be crossed with cultivated potato and require application of other methods for the *Rpi* gene introgression. Recently, 189 potato genotypes, from 20 wild species and cultivated *Solanum tuberosum* from Andigenum and Chilotanum groups, were screened for their resistance against *P. infestans* (Duan et al. 2021). Ten genotypes from five wild species originating in Mexico showed a broad-spectrum resistance to all four *P. infestans* used, suggesting that each of these genotypes contains *Rpi* gene(s) other than *R1*-*R11*. They belong to *S. bulbocastanum* (3 genotypes), *S. cardiophyllum* (4), *S. jamesii* (1), *S. brachycarpum* (1) and *S. trifidum* (1). The other 127 genotypes displayed isolate-specific resistance.

**Table 2:** New sources of late blight resistance in *Solanum* species, where the underlying genes have not been described

Species	Accession	Endosperm Balance Number	Origin	# Tested plants	# Resistant plants	Resistance		References
						*P. infestans* isolate/clonal lineage	Score^b^	
*S. albornozii*	561636	2	Ecuador	5	1	US-23	7–9	Karki et al. (2021b)
*S. agrimonifolium*	545748	2	Mexico	5	3	US-23	7–9	
*S. acaule*	30040	2	Bolivia	NA	NA	NA	7	Bachmann-Pfabe et al. (2019)
	30044			NA	NA	NA	2.7	
	30052			NA	NA	NA	5.9	
*S. albicans*	NA	4	Ecuador	NA	NA	US-23	8.3	Khiutti et al. (2015)
*S. antipovichii*	Buk 59b	NA	Mexico	NA	NA	MP-324	6.8	Zoteyeva et al. (2012)
*S. chomatophilum*	275202	2	Peru	5	5	US-23	7–9	Karki et al. (2021b)
*S. ehrenbergii*	184762	1	Mexico	5	1	US-23	7–9	
	255519			5	2	US-23; NL13316	7–9	
*S. fendleri*	CIP 761921	2	Mexico	48	6	PCO002	NA	Pérez et al. (1999)
	CIP 761923			45	0	PCO002	NA	
	CIP 761926			48	0	PCO002	NA	
*S. gourlayi*	NA	4	Argentina	45^a^	3	NA	6–9	Zoteyeva (2020)
*S. guerreroense*	PI 473088	4	Mexico	NA	NA	MP322	8.0	Zoteyeva et al. (2012)
*S. hougasii*	CIP 761902	4	Mexico	48	12	PCO002	NA	Pérez et al. (1999)
	CIP 761899			48	11	PCO002	NA	
*S. hypacrarthrum*	473477	1	Peru	5	5	US-23	7–9	Karki et al. (2021b)
*S. immite*	NA	4	Peru	NA	NA	US-23	8.4	Khiutti et al. (2015)
*S. iopetalum*	CIP 761928	4	Mexico	48	0	PCO002	NA	Pérez et al. (1999)
	CIP 761923			48	0	PCO002	NA	
	CIP 761926			48	16	PCO002	NA	
*S. kurtzianum*	NA	2	Argentina	82^a^	1	NA	6–9	Zoteyeva (2020)
*S. lesteri*	NA	1	Mexico	NA	NA	US-23	8.6	Khiutti et al. (2015)
*S. megistacrolobum*	35387	2	Bolivia	NA	NA	NA	8.0	Bachmann-Pfabe et al. (2019)
*S. morelliforme*	275222	NA	Mexico	5	3	US-23; NL13316	7–9	Karki et al. (2021b)
	545774			5	2	US-23; NL13316	7–9	
*S. neoantipovichii*	NA	NA	Mexico	20	NA	NA	6.5	Zoteyeva et al. (2012)
*S. neocardenasii*	498129	NA	Bolivia	5	2	US-23; NL13316	7–9	Karki et al. (2021b)
*S. oplocense*	NA	NA	Bolivia	32^a^	2	NA	6–9	Zoteyeva (2020)
*S. oxycarpum*	NA	2	Mexico	2^a^	2	NA	6–9	
*S. palustre*	473401	1	Chile	5	1	US-23	7–9	Karki et al. (2021b)
	558169			5	5	US-23	7–9	
*S. papita* Rydb	Japa, W 273	2	Mexico	18	NA	NA	5.8	Zoteyeva et al. (2012)
*S. papita*	PI 251740			18	NA	NA	6.4	
	PI 251741			12	NA	NA	6.8	
	PI 283105			9	NA	NA	6	
*S. polytrichon* Rydb	GLKS 62.102.6.3	2	Mexico	30	NA	NA	4.6	
*S. polytrichon*	Germany, plt. 102			22	NA	NA	5.9	
	PI 255545			6	NA	NA	7.5	
*S. raphanifolium*	NA	2	Peru	6^a^	1	NA	6–9	Zoteyeva (2020)
*S. sparsipilum*	NA	2	NA	39^a^	10	NA	6–9	
*S. spegazzinii*	NA	2	Argentina	58^a^	7	NA	6–9	
*S. stipuloideum*	498116	1	Bolivia	5	2	US-23	7–9	Karki et al. (2021b)
	498117			5	5	US-23	7–9	
	498118			5	3	US-23	7–9	
	498119			5	5	US-23	7–9	
*S. tarnii*	32871	NA	Mexico	NA	NA	NA	7.2	Bachmann-Pfabe et al. (2019)
*S. vallis-mexici*	NA	NA	Mexico	2^a^	2	NA	6–9	Zoteyeva (2020)
*S. vernei*	NA	2	Argentina	34^a^	26	NA	6–9	

NA, not available

^a^In Zoteyeva (2020), number of accessions

^b^1–9 scale, where 9 is the most resistant

## Structure of *Rpi* genes and their distribution in the potato genome

Most of the plant resistance (*R*) genes are members of a large gene family that encodes nucleotide-binding site and leucine-rich repeat (NB-LRR; NLR) domain-containing proteins (Lozano et al. 2015). On the basis of the structure of NLR proteins, two main groups can be distinguished. The first is the so-called TIR-NB-LRRs (TNLs) with N-terminal domain homologous to the *Drosophila* Toll domain and human interleukin-1 receptor. The second group is non-TIR-NB-LRRs known as CNLs, which contains coiled coil (CC) structure or leucine zipper (LZ) motif in N-terminal region (Ballvora et al. 2002; Sekhwal et al. 2015).

Genome sequencing revealed that the diploid potato clone RH89-039-15 (*S. tuberosum* ssp. *tuberosum*) contains 738 partial or full-length NLR sequences (Bakker et al. 2011). In the potato reference genome DM, 438 out of 40,000 identified genes contain the characteristic NB-LRR domain (Jupe et al. 2012). The use of RenSeq led to an increase in the number of identified NLR in the DM reference genome from 438 to 755 (Jupe et al. 2013). All twelve potato chromosomes contain genes belonging to the CNL and TNL groups, except for chromosomes III and X, on which genes from the TNL group are not found. The majority of NLR genes were found on chromosomes IV (57) and XI (54). The fewest number of NLR genes (3) was found on chromosome III. Moreover, the greatest number of NLR gene clusters is on chromosome IV. There are 4.7 times more CNL genes than TNL genes in the analyzed potato genome (Jupe et al. 2012). Recently, using Illumina HiSeq 2000 technology, 585 NBS domains, including 11 not previously described, were analyzed in 96 potato genomes (Prakash et al. 2020).

To date, nearly 50 *Rpi* genes that are from *Solanum* species have been cloned. Most of the cloned genes belong to the CNL family, but several NLR genes remain unclassified (Table 3). The size and structure of different *Rpi* genes, as well as of different alleles of the same *Rpi* gene, are diverse. Examples of the longest *Rpi* genes include *Rpi-amr1-2307* (7277 bp) from *S. americanum* and *Rpi-blb2* from *S. bulbocastanum* (4858 bp) belonging to the CC-NB-LRR class, and *R1* from *S. demissum* (4102 bp) which is part of LZ-NB-LRR group. The shortest genes include *R2* family members, e.g., *R2* from *S. demissum* (2538 bp), *Rpi-edn1.1* from *S. edinense* (2544 bp), *Rpi-hjt1.1*, *Rpi-hjt1.2* and *Rpi-hjt1.3* from *S. hjertingii* (2544 bp). These genes are located on chromosome IV and are members of the LZ-NB-LRR group. Most of the *Rpi* genes are intron-free. Variation in the size and structure of *Rpi* gene alleles is well described for *Rpi-amr1.* The size of the identified alleles of this gene ranged from 2768 to 7277 bp and the number of introns range from one to four (Table 3). The listed homologues of the *Rpi-amr1* gene have been identified in different species and in different accessions. Molecular cloning of the *Rpi* genes facilitates studies at the molecular level of the control of resistance to potato late blight (Ballvora et al. 2002). The cloned genes can be used in genetic engineering to develop late blight resistant cultivars.

**Table 3:** Sequenced resistance genes against *Phytophthora infestans* (*Rpi* genes)

*Rpi* gene	Class of NB-LRR protein	Accession/patent number	CDS (bp)	Number of introns	References
*R1*	LZ	AF447489.1	4102	2	Ballvora et al. (2002)
*R2*	LZ	FJ536325.1	2538	0	Lokossou et al. (2009)
*R3a*	CC	AY849382.1	3849	0	Huang et al. (2005)
*R3b*	CC	JF900492.1	3582	0	Li et al. (2011)
*R8*	CC	KU530153.1	3738	0	Vossen et al. (2016)
*R9a*	CC	NA	2593	NA	Jo (2013)
*Rpi-abpt*	LZ	FJ536324.1	2538	0	Lokossou et al. (2009)
*Rpi-amr1-1032*	CC	MW345287.1	5120	4	Witek et al. (2021)
*Rpi-amr1-1101*	CC	MW345288.1	5126	4	
*Rpi-amr1-1123*	CC	MW345289.1	5128	4	
*Rpi-amr1-2271*^a^	CC	MW345290.1	2768	1	
*Rpi-amr1-2272*	CC	MW345291.1	5056	4	
*Rpi-amr1-2273*	CC	MW345286.1	4810	3	
*Rpi-amr1-2300*	CC	MW345292.1	5125	4	
*Rpi-amr1-2307*	CC	MW345293.1	7277	4	
*Rpi-amr1-3408*	CC	MW345294.1	3749	3	
*Rpi-amr1-3409*	CC	MW345295.1	5130	4	
*Rpi-amr3*	CC	KT373889.1	2664	0	Witek et al. (2016)
*Rpi-ber1.1_94-2031*	CC	MW410790.1	3909	0	Monino-Lopez et al. (2021)
*Rpi-ber1.2_493-7*^a^	CC	MW410793.1	3912	0	
*Rpi-ber1.3*	CC	MW410798.1	3912	0	
*Rpi-ber1.4*	CC	MW410802.1	3898	0	
*Rpi-blb1*	CC	AY426259.1	3592	1	Van der Vossen et al. (2003)
*Rpi-blb2*	CC	DQ122125.1	4858	2	Van der Vossen et al. (2005)
*Rpi-blb3*	LZ	FJ536346.1	2544	0	Lokossou et al. (2009)
*Rpi-bt*	NA	FJ188415.1	3379	1	Oosumi et al. (2009)
*Rpi-chc1.1*	CC	MW383255.1	3909	0	Monino-Lopez et al. (2021)
*Rpi-chc1.2*^a^	CC	MW410797.1	3912	0	
*Rpi-edn1.1*	LZ	GU563963.1	2544	0	Champouret (2010)
*Rpi-edn1.2*	NA	NA	NA	NA	
*Rpi-edn2*	CC	US20140041072A1	2593	NA	De Vetten et al. (2014)
*Rpi-hcb1.1*	CC	NA	NA	NA	Aguilera-Galvez et al. (2020)
*Rpi-hcb1.2*	CC	NA	NA	NA	
*Rpi-hjt1.1*	LZ	GU563971.1	2544	0	Champouret (2010)
*Rpi-hjt1.2*	LZ	GU563972.1	2544	0	
*Rpi-hjt1.3*	LZ	GU563973.1	2544	0	
*Rpi-mcd1*	NA	NA	NA	NA	Lokossou (2010)
*Rpi-mcq1*	CC	WO2009013468A2	NA	NA	Jones et al. (2009)
*Rpi-nrs1*	CC	WO2009013468A2	NA	NA	
*Rpi-pta1*	NA	EU884422.1	3592	1	Vleeshouwers et al. (2008)
*Rpi-snk1.1*	LZ	GU563975.1	2544	0	Champouret (2010)
*Rpi-snk1.2*	LZ	GU563976.1	2535	0	
*Rpi-sto1*	NA	EU884421.1	3592	1	Vleeshouwers et al. (2008)
*Rpi-sto2*	CC	NA	NA	NA	Champouret (2010)
*Rpi-tar1.1_852-5*	CC	MW390807.1	3912	0	Monino-Lopez et al. (2021)
*Rpi-tar1.3*	CC	MW410799.1	3912	0	
*Rpi-vnt1.1*	CC	FJ423044.1	2676	0	Foster et al. (2009)
*Rpi-vnt1.2*	CC	FJ423045.1	2718	0	
*Rpi-vnt1.3*	CC	FJ423046.1	2718	0	
*Ph-3*	CC	KJ563933.1	2556	0	Zhang et al. (2014)

^a^Non-functional/susceptible homolog

bp, base pairs; CDS, coding sequence; LZ, leucine zipper motif; CC, coiled coil motif; NA, not available

Plant *R* genes encode proteins that directly or indirectly detect effector proteins introduced by pathogens (Sekhwal et al. 2015). This leads to the activation of effector-triggered immunity (ETI) and results in reactive oxygen species (ROS) production, callose deposition, and programmed cell death through the hypersensitive response (HR) (Turnbull et al. 2019). The mechanism of potato *Rpi* gene activation by *P. infestans* effectors is poorly understood. Studies conducted on *Arabidopsis thaliana* show that the conformation of NLR proteins may influence their function. The *A. thaliana* NLR protein Peronospora parasitica 1 protein (RPP1), which recognizes the ATR1 effector from *Peronospora parasitica*, remains in an inactive form in the absence of an effector in the cell environment. Binding of the effector to the LRR domain leads to oligomerization and activation of the RPP1 protein (Schreiber et al. 2016). Another *A. thaliana* NLR protein, ZAR1, in an inactive form, forms a multicomponent complex with resistance-related kinase 1 (RKS1) (Wang et al. 2019). Inactive ZAR1-RKS1 complex is activated by the effector AvrAC from *Xanthomonas campestris.* AvrAC uridylates the PBL2 kinase to produce PBL2^UMP^. Interactions between PBL2^UMP^ and the inactive ZAR1-RKS1 complex then lead to conformational changes and the formation of the active pentameric ZAR1 resistosome (ZAR1-RKS1-PBL2^UMP^). The active resistosome has a funnel-shaped structure required for AvrAC-induced ZAR1 plasma membrane association, cell death, and resistance to *X. campestris*. Nonetheless, how widespread these models are and whether the mechanism of action is the same in potato are unknown (Wang et al. 2019). Activation of *Rpi* genes may also depend on external factors. Rpi-vnt1.1 requires light to confer resistance against *P. infestans*. In the dark, plants produce shortened chloroplast protein glycerate 3-kinase (GLYK), which does not bind Avrvnt1 and that results in a lack of activation of the Rpi-vnt1.1 protein (Gao et al. 2020).

## Arms race

The host immune response against pathogen invasion can be summarized by the zig-zag model, which involves two steps. The first step relates to the detection of the conserved pathogen-associated molecular pattern (PAMP) which triggers PAMP-triggered immunity (PTI). To avoid PTI, the pathogen secretes effector proteins into the host cells to disrupt the immune response. Effectors are detected by the host's NLRs, leading to activate more robust and faster response termed ETI, which represents the second level of activation the host immune response. Interactions between *R* genes and effectors represent host–pathogen molecular co-evolution when effectors evolve to evade detection and R proteins evolve to establish or retain detection (Hein et al. 2009; Naveed et al. 2020).

During the pathogenesis of *P. infestans*, a key step is the formation of haustorium in potato tissue through which the pathogen secretes effectors. These proteins manipulate and alter the host's immune response to promote infection. Genes encoding pathogen effectors that induce *R* gene response are defined as avirulence (*Avr*) genes (Qutob et al. 2006). Cytoplasmic effectors secreted by *P. infestans* can be divided into two classes, CRN (crinkling, necrosis) and RxLR effectors. The effectors of RxLR type possess arginine-any amino acid residue-leucine–arginine motifs in N-terminal region. All known *P. infestans* effectors, which are recognized by the products of corresponding potato *Rpi* genes, belong to the RxLR class (Martynov and Chizhik 2020). The RxLR effectors contain the highly conserved N-terminal RxLR motif involved in the translocation of *P. infestans* effector proteins into plant cells, and the heterogeneous C-terminal region that can be recognized by plant *R* gene products (Dou et al. 2008).

The function of the *P. infestans* effector in the infection process has been defined for only a few examples. Avr2, by interacting with members of the BRI1-suppressor 1-like family proteins (BSL1, BSL2 and BSL3) from potato, inhibits the activity of the oomycete infestin 1 (INF1); as a result, programmed cell death does not occur (Turnbull et al. 2019). Avr3 inactivates the host ubiquitin E3 ligase CMPG1, leading to programmed cell death inhibition (Bos et al. 2010). The effector Avr3a-like, by stabilizing host cinnamyl alcohol dehydrogenase 7 (CAD7), limits the activation of defense mechanisms, such as callose deposition, the ROS burst and WRKY33 expression (Li et al. 2019).

In the genome of *P. infestans*, 563 effector genes with the RxLR motif have been identified (Haas et al. 2009). For 15 of them, the respective potato *Rpi* genes have been identified (Table 4). According to the gene-for-gene concept, specific pathogen effectors activate corresponding host plant R proteins (Flor 1971). However, some effectors can be recognized by products of multiple *Rpi* genes, or vice versa, a specific *Rpi* gene product can detect multiple effectors from the same or different *Phytophthora* pathogens. The Avr2 effector can be recognized by not only R2 protein from *S. demissum* but also by Rpi-blb3 from *S. bulbocastanum*, Rpi-mcq1 from *S. mochiquense,* Rpi-hcb1.1 and Rpi-hcb1.2 from *S. huancabambense* (Aguilera-Galvez et al. 2018, 2020)*.* The *Rpi* genes encoding these proteins are located on different chromosomes, *R2* and *Rpi-blb3* on chromosome IV, *Rpi-mcq1*, *Rpi-hcb1.1* and *Rpi-hcb1.2* on chromosome IX (Table 1). Rpi-amr1 from *S. americanum* recognizes the effector Avramr1 from *P. infestans* but also the Avramr1 homologues from *Phytophthora parasitica* and *Phytophthora cactorum* (Witek et al. 2021). Likewise, recognition of Avramr3 by Rpi-amr3 from *S. americanum* activates resistance against not only *P. infestans* but also against other economically important *Phytophthora* pathogens, including *P. parasitica* and *Phytophthora palmivora* (Lin et al. 2021). The authors suggest that *Rpi-amr1* and *Rpi-amr3* provide non-host type resistance to multiple *Phytophthora* pathogens in *S. americanum* (Lin et al. 2021; Witek et al. 2021). In some cases, different alleles from the same *Rpi* gene can recognize different effectors which belong to the same RxLR family (Monino-Lopez et al. 2021). Protein products of *Rpi-chc1.1* and *Rpi-chc1.2* which are allelic variants recognize two different effectors within the same effector class Avrchc1.1 and Avrchc1.2, respectively. The LRR domain is involved in the recognition of cognate effector and changes in its structure may lead to the loss of the ability to recognize the effector or to shift the recognition ability from one effector to another. The exchange of the LRR domain in the chimeric receptors changed the recognition spectrum of the Avrchc1.1 to Avrchc1.2 (Monino-Lopez et al. 2021).

**Table 4:** *Phytophthora infestans* genes encoding RxLR effectors and corresponding resistance genes (*Rpi* genes) in host plants

*P. infestans* effectors	Corresponding *Rpi* gene(s)	References
*Avr1*	*R1*	Van der Lee et al. (2001)
*Avr2*	*R2; Rpi-mcq1; Rpi-blb3;* *Rpi-hcb1.1; Rpi-hcb1.2*	Aguilera-Galvez et al. (2018); Aguilera-Galvez et al. (2020)
*Avr3a*	*R3a*	Armstrong et al. (2005)
*Avr3b*	*R3b*	Rietman et al. (2012)
*Avr4*	*R4*	Van Poppel et al. (2008)
*Avr8*	*R8*	Vossen et al. (2016)
*Avrblb1(ipiO)*	*Rpi-blb1*	Song et al. (2003)
*Avrblb2*	*Rpi-blb2*	Van der Vossen et al. (2005)
*Avrvnt1*	*Rpi-vnt1.1*	Pais et al. (2018)
*AvrSmira1*	*Rpi-Smira1*	Rietman et al. (2012)
*AvrSmira2*	*Rpi-Smira2*	
*Avrchc1.1*	*Rpi-chc1.1; Rpi-ber1.1*	Monino-Lopez et al. (2021)
*Avrchc1.2*	*Rpi-chc1.2; Rpi-ber1.2*	
*Avramr1*	*Rpi-amr1*	Witek et al. (2021)
*Avramr3*	*Rpi-amr3*	Lin et al. (2021)

To date, several mechanisms have been identified that allow effectors to avoid recognition by corresponding host R proteins. The products of such unrecognized alleles act as virulence factors. *P. infestans* virulence can arise in multiple ways (Huang et al. 2019). The simplest one involves point mutations, e.g., the *P. infestans* effector *Avr3a*. In tested *P. infestans* populations, two alleles of *Avr3a* differing at the protein level by only two amino acids can be distinguished. *Avr3a*^*KI*^ activates the potato resistance gene *R3a,* leading to a HR, while *Avr3a*^*EM*^ is not recognized by the product of the *R3a* gene, leading to infection (Armstrong et al. 2005). Isolates with truncated Avr4 protein resulting from a frameshift generated by two single deletions are not detected by the product of the *R4* gene in potato. Functional analysis shows that two single deletions do not affect the elicitor activity of the Avr4 protein (Van Poppel et al. 2008). *P. infestan*s isolates that possess the *Avr1* homologue *Avr1-like* (A-L) do not induce a resistance response in *R1* potato. The sequence of the Avr1-like (A-L) effector is in 82% identical to that of the Avr1 protein but is truncated by the T region at the C-terminal end of Avr1 (Du et al. 2018). Due to changes in expression regulation, the *Avrvnt1* effector is not detected by *Rpi-vnt1* plants (Stefańczyk et al. 2017; Pais et al. 2018). Expression of *Avrchc1.2* effector is rapidly downregulated in the first hours after inoculation with *P. infestans* isolates, which explains why the presence of Rpi-chc1.2 in the potato plants does not provide resistance to late blight (Monino-Lopez et al. 2021). The Rpi-blb1 protein recognizes ipiO (Avrblb1) effectors from classes I and II, resulting in a HR. Effectors from class III, i.e., ipiO4, are not recognized by the Rpi-blb1 protein and inhibit HR caused by classes I and II of the effector (Champouret 2010). Potentially, the effectors that are essential for infection could not be mutated without a fitness cost and loss of pathogenicity. R proteins recognizing such essential effectors would likely provide broad-spectrum and durable resistance. *P. infestans Avr3a*, especially virulent allele *Avr3a*^*EM*^, may be an example of an essential effector since this gene is conserved among diverse *P. infestans* strains and is highly expressed at the early stage of infection (Yin et al. 2017). Further searching and functional characterization of conserved effectors and corresponding *Rpi* genes may inform strategies for obtaining durable late blight resistance.

The adaptation of potato to the continuous evolution of the pathogen is through the diversification of *R* genes by recombination, gene conversion, duplication and/or selection (Jupe et al. 2012). While some of the *S. demissum Rpi* genes were found to be race-specific and rapidly became ineffective, the following genes have been described as providing a broad-spectrum of resistance against *P. infestan*s: *Rpi-blb1, Rpi-blb2* and *Rpi-blb3* from *S. bulbocastanum*; *R8* and *R9* from *S. demissum* and *Rpi-vnt1.1* from *S. venturii* (Vleeshouwers et al. 2011; Vossen et al. 2016). However, these genes have not yet been widely introduced into potato cultivars, in part because of crossing barriers. This continuous co-evolution of pathogen effectors and plant *R* genes represents a so-called arms race between plants and pathogens (Khavkin 2015).

## *Rpi* genes in potato cultivars and breeding lines

Breeding potato cultivars with resistance genes against *P. infestans* gives opportunities to limit the use of fungicides (Haverkort et al. 2016). However, breeders often do not know what *R* genes are present in existing potato cultivars and which of them are effective against local *P. infestans* populations. Some potato cultivars show moderate or high levels of resistance to late blight, but the basis of their resistance remains unknown. Various methods, including PCR, effectoromics, transcriptomics, single nucleotide polymorphism (SNP) array genotyping or dRenSeq, have been used to determine which *Rpi* genes are present in potato cultivars (Table 5). Frequently, a combination of more than one method was used.

**Table 5:** Resistance genes against *Phytophthora infestans* (*Rpi* genes) present in potato cultivars

Year of registration	Cultivar	*Rpi* gene	Methods of detection	References
Europe				
2014	Alouette	*Rpi-vnt1.3; R3a; R3b*	dRenSeq	Armstrong et al. (2019)
1925	Alpha^a^	*R8*	PCR markers; DLA; field trials; pedigree	Rogozina et al. (2021)
NA	Avrora	*RB/Rpi-blb1; Rpi-sto1*	PCR markers; sequencing	Antonova et al. (2018)
1910	Bintje^a^	*Rpi-vnt1.3*	PCR markers; DLA; field trials; pedigree	Rogozina et al. (2021)
2004	Biogold	*Rpi-abpt*	NA	Park et al. (2005b)
2008	Bionica	*Rpi-blb2; Rpi-abpt; R3a; R3b*	dRenSeq; pedigree	Haverkort et al. (2009); Armstrong et al. (2019)
1983	Bzura	*R1; R2-like*	PCR markers; field trials; DLA; sequencing; pedigree	Gebhardt et al. (2004); Plich et al. (2015)
1973	Cara	*R1; R3a; R3b*	dRenSeq	Armstrong et al. (2019)
1941	Craigs Snow White	*R1*		
1961	Dorita	*R3b*	PCR markers; DLA; field trials; pedigree	Brown-Donovan et al. (2021)
1892	Eersteling^a^	*R8*	PCR markers; DLA; field trials; pedigree	Rogozina et al. (2021)
1996	Elizaveta^a^	*R1; R3a; R3b; Rpi-blb1*		
1982	Escort	*R1; R2; R3a; R3b*		
2018	Gardena	*Rpi-phu1*	PCR markers; DLA; pedigree	Stefańczyk et al. (2020)
NA	Gloria	*R2; R3a; R3b*	PCR markers; DLA; field trials; pedigree	Rogozina et al. (2021)
1999	Innovator	*R1; R2-like; R3a; R3b*	dRenSeq	Armstrong et al. (2019)
1908	Jubel	*R1; R2; R8; Rpi-blb2; Rpi-vnt1.3*	PCR markers; DLA; field trials; pedigree	Rogozina et al. (2021)
NA	Nayada	*R1; R2; Rpi-blb3; R8; Rpi-blb2*		
NA	Negr^a^	*R2*		
NA	Ognivo	*RB/Rpi-blb1; Rpi-sto1*	PCR markers; sequencing	Antonova et al. (2018)
1952	Pentland Ace^a^	*R3a; R3b*	dRenSeq	Armstrong et al. (2019)
1961	Pentland Dell	*R1; R3a; R3b; Rpi-abpt*		
1994	Picasso	*R1; R3a; R3b*		
1976	Pirola	*Rpi-phu1*	PCR markers; DLA; field trials; pedigree	Brown-Donovan et al. (2021)
NA	Priekul'skij rannij	*R8; Rpi-blb1*		Rogozina et al. (2021)
1926	Robijn	*R2*		
NA	Sárpo Axona	*R3a; R3b; Rpi-vnt1.3*		
2003	Sárpo Mira	*R3a; R3b; R4; Rpi-Smira1; Rpi-Smira2*	Effectoromics; DLA	Rietman et al. (2012)
1968	Spunta	*R1*	dRenSeq	Armstrong et al. (2019)
1991	Stirling	*R1; R3b*	PCR markers; DLA; field trials; pedigree	Brown-Donovan et al. (2021)
NA	Svitanok kievskij	*R2; Rpi-blb3; R3a; R3b; R8; Rpi-blb1*		Rogozina et al. (2021)
2006	Toluca	*Rpi-blb2*	dRenSeq; pedigree	Haverkort et al. (2009); Armstrong et al. (2019)
1988	Torridon	*R1; R3b*	PCR markers; DLA; field trials; pedigree	Brown-Donovan et al. (2021)
North America				
1970	Abnaki^a^	*R1*	PCR markers; DLA; field trials; pedigree	Brown-Donovan et al. (2021)
NA	Atzimba	*R8; Rpi-blb1; Rpi-blb2*		Rogozina et al. (2021)
1867	Early Rose^a^	*Rpi-blb2; Rpi-vnt1.3*		
1999	Jacqueline Lee	*R8*	Long-range PCR; sequencing	Vossen et al. (2016)
2009	Missaukee			
2015	Payette Russet	*R2*	SNP array genotyping; DLA; KASP markers	Karki et al. (2021a)
1950	Pungo	*R1*	PCR markers; DLA; field trials; pedigree	Brown-Donovan et al. (2021)
NA	Saginaw Chipper	*R2*		
1980	Tollocan	*R3b*		
2006	Yukon Gem			
Asia				
NA	Cooperation 88 (C88)	*R1;R2;R3a; Rpi-blb1; Rpi-blb2; Rpi-vnt1*	RNA-seq; DLA	Hao et al. (2018)
NA	Longshu 7	*R3a; Rpi-vnt1.1*	PCR markers; agroinfiltration assay; pedigree	Elnahal et al. (2020)
NA	PB-06	*R8*	Long-range PCR; sequencing	Vossen et al. (2016)
NA	Qingshu 9	*R3a; R4; R8*	PCR markers; agroinfiltration assay; pedigree	Elnahal et al. (2020)
1961	Rishiri	*R1*	Pedigree	Akino et al. (2014)
NA	S-60	*R8*	Long-range PCR; sequencing	Vossen et al. (2016)
1976	Toyoshiro	*R1*	Pedigree	Akino et al. (2014)
1958	Yoraku	*R4*		

^a^PCR marker detected, but the cultivar is described in literature as susceptible to late blight

dRenSeq, diagnostic resistance gene enrichment sequencing; DLA, detached leaf assay; SNP, single-nucleotide polymorphism; NA, not available

Analysis of 600 potato cultivars from Europe, Asia, and South America by PCR using gene-specific primers, allowed to detect *R1* in 135 potato genotypes (Gebhardt et al. 2004). Using gene-specific markers, it was possible to confirm the presence of the *R1* and *R2-like* genes in the Polish cultivar Bzura showing a high level of field resistance (Plich et al. 2015). The Mastenbroek potato late blight differential set is a group of 11 potato genotypes (Ma*R1*-Ma*R11*) expected to contain 11 individual *S. demissum Rpi* genes (Mastenbroek 1952). However, studies using *Rpi* gene-specific markers and agroinfiltration assay showed that differential plants harbor more than one *Rpi* gene. Ma*R8* and Ma*R9* plants, which have a broad-spectrum resistance in both the field and the greenhouse, contain four (*R3a*, *R3b*, *R4*, *R8*) and seven (*R1*, *Rpi-abpt*, *R3a*, *R3b*, *R4*, *R8*, *R9*) *Rpi* genes, respectively (Kim et al. 2012; Zhu et al. 2015). *R1* was additionally found in Ma*R5* and Ma*R6* genotypes. These findings are consistent with those of Trognitz and Trognitz (2007), who found *R1* in the *R5*, *R6* and *R9* plants also within the Scottish Black’s differential set. The *Rpi-vnt1* gene from *S. venturii* and *Rpi-phu1* from *S. phureja* were mapped to the same region on the potato chromosome IX and the nucleotide sequences of both genes are identical (Śliwka et al. 2006; Foster et al. 2009). The *Rpi-vnt1.1* confers resistance to a wide range of *P. infestans* strains, except isolates EC1 and EC3626 from Ecuador (Foster et al. 2009; Witek et al. 2021). The presence of the *Rpi-vnt1.1* gene has been confirmed in Dutch cultivar Alouette and in Polish cultivar Gardena using the PCR marker phu1_2069 (Stefańczyk et al. 2020). The *Rpi-vnt1.1* have been also found in six other cultivars (Table 5). It is worth underlining that PCR markers designed on the basis of gene sequence may display low specificity. Detection of the *Rpi-vnt1* gene in late blight susceptible cultivars, including Bintje and Early Rose, is most likely due to use of non-specific markers and the detection of non-functional homologues (Rogozina et al. 2021). Detection of the presence *Rpi* genes in potato cultivar with the use of PCR markers requires additional methods confirming resistance to *P. infestans*, as it is prone to false-positive results.

Another strategy is the effectoromics approach, where effectors are functionally tested in potato germplasms for their response to cognate *R* gene using agroinfiltration assay (Domazakis et al. 2017). More than 200 predicted RxLR effectors selected from *P. infestans* genome sequence were used in an agroinfiltration test, which allowed to detect five effective *Rpi* genes (*R3a*, *R3b*, *R4*, *Rpi-Smira1*, *Rpi-Smira2*) in the Sárpo Mira cultivar characterized by a high level of field resistance (Rietman et al. 2012). Four of them were pyramided qualitative *Rpi* genes. The remaining one, *Rpi-Smira2*, provides a quantitative field resistance, and its presence in potato cultivar can only be detected in field tests. *R8* with nucleotide sequence identical to that of *Rpi-Smira2* can be found also in the resistant potato cultivars Jacqueline Lee, Missaukee, PB-06 and S-60, by long-range PCR (Vossen et al. 2016). Agroinfiltration with ten *P. infestans* effectors revealed that Avr4 and Avr8 effector induce HR in potato cultivar Qingshu9, Avrvnt1.1 induces HR in Longshu7, Avr3a^EM^ effector (i.e., virulent allele of Avr3a^KI^) induces HR in cultivars Qingshu9 and Longshu7 (Elnahal et al. 2020). Screening with over 50 different *P. infestans* RxLR effectors has shown a specific response to Avr2, which confirms that SW93-1015 contains a functional homologue of the *R2* gene. Out of *R2* gene homologues cloned from SW93-1015, one encoded a protein identical to Rpi-abpt. Transgenic potato cultivar Désirée with this gene was resistant to *P. infestans* (Lenman et al. 2016).

Analysis of the transcriptome of the Chinese cultivar Cooperation 88 (C88), which has been characterized as displaying durable late blight resistant for 20 years, revealed the presence of multiple *Rpi* genes (Hao et al. 2018). This cultivar is highly resistant to two super virulent *P. infestans* strains, IPO 428–2 and XA-4. Within 5 days of inoculation with XA-4, a change in the expression of *Rpi* genes was noted. These genes can be classified as *R1*, *R2*, *R3a*, *Rpi-blb1*, *Rpi-blb2* and *Rpi-vnt1* homologues (Hao et al. 2018).

SNP array genotyping can also be used to detect the *R* genes in the cultivated potato (Karki et al. 2021a). F1 population containing 79 progeny clones derived from crossing Payette Russet with A0012–5 was screened for resistance to the US-23 genotype of *P. infestans* in detached leaf assay. Linkage mapping using markers from the potato SNP array confirmed the presence of a single resistant gene on the short arm of chromosome IV of cultivar Payette Russet, in the same locus as that for *R2*, *Rpi-abpt*, and *Rpi-blb3*. Using the primers for *Rpi-blib3*, a PCR product of the expected size (~ 2500 bp) was obtained and sequenced. The *Rpi* gene allele from Payette Russet is identical to the *Rpi-abpt* sequence except for a synonymous C to T substitution at position 87 (Karki et al. 2021a).

A new tool used for genetic mapping, searching, and testing the functionality of resistance genes in cultivars and breeding lines is dRenSeq (Armstrong et al. 2019). dRenSeq has been used to identify and validate all currently known NLRs effective against potato virus X, the potato cyst nematode *Globodera pallida* and *P. infestans*. Screening by dRenSeq for the presence of 22 functional *Rpi* genes in 11 potato cultivars and one late blight differential line 2573 led to the identification of one to seven *Rpi* genes in each tested genotype (Table 5). Single *Rpi* genes were found in cultivars Craigs Snow White, Spunta and Toluca. Seven *Rpi* genes, i.e., *R1*, *R1*^*ΔT4109*^, *R3a*, *R3b*^*G1696/G3111*^, *R8*, *R9a*, and *Rpi-abpt*^*T86*^, have been identified in the differential line 2573 (Armstrong et al. 2019).

## Genetic improvement for durable *P. infestans* resistance

The introgression of *Rpi* genes into susceptible potato cultivars is limited by long breeding cycles and the high level of heterozygosity across the potato genome (Jo et al. 2014). An attempt to introgress the *Rpi* genes from *S. bulbocastanum* began in 1959. In the first step, a cross was made between *S. bulbocastanum* (B, 2x) bearing *Rpi-blb2* and *S. acaule* (A, 4x) to obtain an AB (3x) plants, which after polyploidisation to the hexaploid level, was crossed with *S. phureja* (P, 2x) resulting in ABP (4x) material. Successive rounds of bridge crosses between ABP (4x) and *S. tuberosum*, led to produce ABPT plants which, after three backcrossing to *S. tuberosum*, eventually led to registration in 2006 and 2008 of two *P. infestans* resistant cultivars Toluca and Bionica (Haverkort et al. 2016). Another example of the introduction of *Rpi* genes into the potato gene pool is the 11-year-long project Bioimpuls, which resulted in the development of true seed population with single or multiple *Rpi* genes against late blight through classical breeding (Keijzer et al. 2021). In this project, three groups of sources of resistance to *P. infestans* were distinguished. The first group includes cultivars and advanced breeding clones containing the *R8, Rpi-cap1, Rpi-chc1, Rpi-vnt1* and *Rpi-blb2* genes that is ready for the commercial crossing. The second group includes breeding clones with *R9* and *Rpi-edn2* genes which require one or two rounds of backcrossing. The third group is potato wild species that have not been used so far, including *S. brachycarpum*, *S. bukasovii*, *S. iopetalum*, *S. multiinterruptum*, and *S. sucrense.* This group needs two or three additional rounds of backcrossing to be used for commercial crosses (Keijzer et al. 2021).

Different approaches have been developed to overcome crossing barriers and to shorten the time for introducing *Rpi* genes into susceptible cultivars, including the use of somatic hybrids, hybrid breeding and genetic engineering. To transfer late blight resistance from *S. michoacanum* to the gene pool of cultivated potato, somatic hybridization and backcrossing (BC) have been used (Smyda et al. 2013; Smyda-Dajmund et al. 2017). The genetic composition of the obtained somatic hybrids was analyzed using diversity array technology (DArT) (Smyda-Dajmund et al. 2016). Using somatic hybridization, crossing and backcrossing, four *Rpi* genes (*Rpi-blb1, Rpi-blb3, R3a*, *R3b*) were introduced into cultivated potato (Rakosy-Tican et al. 2020). The presence of these genes in the back-crossed progeny (BC_1_ and BC_2_) was confirmed via gene-specific markers. In addition, the functionality of *Rpi-blb1*, *Rpi-blb3*, *R3a* and *R3b* was confirmed via agroinfiltration with the corresponding *Avr* effectors (*Avrblb1*, *Avr2, Avr3a, Avr3b*).

Another method to facilitate transfer of resistance to late blight to potato cultivar is hybrid breeding. This method enables obtaining plants with single or pyramided *Rpi* genes without disrupting the genetic composition of the parental breeding lines that have good agronomic performance. Su et al. (2020) described the introgression of *Rpi* genes into homozygous diploid potato. First, four different *Rpi* genes, which were derived from *S. avilesii*, *S. tarijense*, *S. venturii* and *S. chacoense*, were introduced in three highly homozygous (e.g., with homozygosity scores as 88%, 88% and 79%) diploid potato breeding lines via marker-assisted introgression. After two backcrosses supported with marker selection and one selfing, parents with the homozygous resistance allele were produced and were used for crossing. The two backcrossing steps ensured the removal of most of the genome of the donor wild species parent, and the selfing step helped to remove any remaining unwanted introgressions. The hybrids were made by crossing two homologous parents, each having a different *Rpi* gene. Finally, hybrids with single *Rpi* gene (*Rpi-avl1*, *Rpi-tar1*, *Rpi-vnt1.1*, *Rpi-chc1.1*) and hybrids with combination of two *Rpi* genes (*Rpi-avl1* and *Rpi-chc1*, *Rpi-avl1* and *Rpi-tar1*, *Rpi-avl1* and *Rpi-vnt1.1*, *Rpi-tar1* and *Rpi-vnt1.1*, *Rpi-vnt1.1* and *Rpi-chc1*) were obtained*.* The hybrids were tested for resistance to *P. infestans* in three separate field trials. The hybrids with two resistance genes were more resistant compared to the ones with the respective single *Rpi* gene. Hybrid breeding with the use of existing elite material and marker-assisted introgression allows obtaining resistant plants in a relatively short time (Su et al. 2020).

An alternative approach involves genetic engineering, which significantly shortens the long time to introgress resistance genes through breeding cycle for tetraploid potato plants (Van Esse et al. 2020). One such method is cisgenesis, i.e., the introduction of genetic material from the same species or from a crossable species (Hou et al. 2014). Transformed potato cultivars obtained by genetic engineering are shown in Table 6. Single *Rpi* genes have been introduced into several potato cultivars. *Rpi-vnt1.1* or *Rpi-sto1* have been introduced separately into the cultivars Atlantic, Bintje and Potae9 (Jo et al. 2014). The obtained transgenic plants were evaluated for late blight resistance in detached leaf assay and agroinfiltration assay using five *P. infestans* isolates. Transgenic Atlantic, Bintje and Potae9 with *Rpi-sto1* were resistant to all tested isolate except pic99189. Transgenic Atlantic and Bintje with *Rpi-vnt1.1* gene were resistant to all tested isolate except EC1 (Jo et al. 2014). *Rpi-vnt1.1* and *Rpi-mcq1* have been transformed separately into the cultivar Désirée and tested in field experiment (Jones et al. 2014). All transgenic plants with the *Rpi-mcq1* gene were susceptible to late blight. Transformed Désirée plants with the *Rpi-vnt1.1* gene remain fully resistant to *P. infestans* or have reduced disease severity compared to susceptible controls (Jones et al. 2014). In another study, whole-plant resistance assays were carried out in the confined biosafety greenhouse to evaluate the late blight resistance of the Désirée plants transformed with *Rpi-vnt1* (Roman et al. 2017). Unexpectedly, 5 out of 52 transgenic events showed resistance to two Peruvian *P. infestans* isolates belonging to the EC-1 lineage. As reported previously, a different isolate of the EC-1 lineage (isolate EC1 from Ecuador, the only one tested from the lineage), in which the cognate effector gene *Avrvnt1* was not expressed, was able to break the resistance conditioned by the *Rpi-vnt1* gene in transformed Désirée plants based on a detached leaf assay (Foster et al. 2009; Pel et al. 2009. The authors inferred the EC-1 isolates used in Peru may differ in virulence within the EC-1 lineage (Roman et al. 2017).

**Table 6:** Resistance genes against *Phytophthora infestans* (*Rpi* genes) transferred to potato cultivars by genetic engineering

*Rpi* gene(s)	Transformed cultivar	Assessment of resistance		References
		Laboratory assay	Field trials	
*Rpi-vnt1.1*	Atlantic	R	NA	Jo et al. (2014)
	Bintje	R	NA	
	Potae9	R	NA	
*Rpi-sto1*	Atlantic	R	NA	
	Bintje	R	NA	
	Potae9	R	NA	
*Rpi-vnt1.1*	Désirée	NA	R	Jones et al. (2014)
*Rpi-mcq1*	Désirée	NA	S	
*Rpi-blb2*	Désirée	R	NA	Orbegozo et al. (2016)
*Rpi-sto1*	Désirée	R	R	Haesaert et al. (2015)
*Rpi-vnt1.1*	Désirée	R	R	
*Rpi-blb3; Rpi-vnt1; Rpi-sto1*	Désirée	R	R	
*Rpi-blb3; Rpi-sto1*	Désirée	NA	R	Haverkort et al. (2016)
*Rpi-vnt1; Rpi-chc1*	Désirée	NA	R	
*Rpi-vnt1; Rpi-sto1*	Désirée	NA	R	
*Rpi-blb3; Rpi-vnt1; Rpi-sto1*	Désirée	NA	R	
*Rpi-vnt1.1*	Désirée	R	NA	Roman et al. (2017)
*RB; Rpi-blb2; Rpi-vnt1.1*	Désirée	NA	R	Ghislain et al. (2019)
	Victoria	NA	R	
*RB; Rpi-blb2; Rpi-vnt1.1*	Tigoni	R	NA	Webi et al. (2019)
	Shangi	R	NA	
*Rpi-ber1.1_94-2031*	Désirée	R	NA	Monino-Lopez et al. (2021)
*Rpi-tar1.1_852-5*	Désirée	R	NA	
*Rpi-chc1.1*	Désirée	R	NA	
*Rpi-amr1-2272*	Maris Piper	R	NA	Witek et al. (2021)
*Rpi-amr1-2273*	Maris Piper	R	NA	

R, resistant; S, susceptible; NA, not available

The resistance provided by a single *Rpi* gene can be quickly overcome by an adapted *P. infestans* strain. A promising breeding approach involves pyramiding several different *Rpi* genes in one potato cultivar. In 2006, a Durable Resistance in potato against *Phytophthora* (DuRPh) project has been initiated at Wageningen University and aimed at developing durable resistance in existing potato cultivars by pyramiding *Rpi* genes via cisgenesis (Haverkort et al. 2009, 2016). Transgenic Désirée potato plants with two *Rpi* genes (*Rpi-blb3:sto1, Rpi-vnt1:ch1* or *Rpi-vnt1:sto1*) and with three *Rpi* genes (*Rpi-blb3:vnt1:sto1*) have been produced. These plants have not been infected by *P. infestans* during the two-year field tests (Haverkort et al. 2016). Transgenic potato cultivar Désirée with three *Rpi* genes (*Rpi-blb3*, *Rpi-vnt1.1* and *Rpi-sto1*) was obtained also by Haesaert et al. (2015). Plants with these genes showed complete resistance to late blight during two-year field trials in Belgium and the Netherlands (Haesaert et al. 2015). In another study, successful stacking of *RB* and *Rpi-blb2* from *S. bulbocastanum* and *Rpi-vnt1.1* from *S. venturii* in the susceptible potato cultivars Désirée and Victoria resulted in complete resistance to late blight (Ghislain et al. 2019). Compared with those with a single *Rpi* gene (*RB*, *Rpi-blb2* or *Rpi-vnt1.1*), transgenic plants with three *Rpi* genes showed a significantly higher level of resistance. In three-year field trials involving transgenic plants in southwestern Uganda, no isolate of *P. infestans* that could overcome the resistance provided by the three *Rpi* genes was found (Ghislain et al. 2019). The same three *Rpi* genes, *RB, Rpi-blb2* and *Rpi-vnt1.1*, were stacked in two popular Kenyan potato cultivars Tigoni and Shangi (Webi et al. 2019).

The level of resistance to late blight in transgenic plants not only is dependent on the recognition spectrum and activity of *Rpi* gene(s), but also can depend on the genetic background of the recipient genotype (Shandil et al. 2017). Potato F1 progenies, obtained from crossing transgenic cultivar Katahdin carrying an *RB* gene with non-transgenic susceptible cultivar Kufri Bahar or resistant cultivar Kufri Jyoti, were screened for resistance to late blight by whole plants assay. The cultivar Kufri Jyoti is late blight resistant, with resistance inherited from *S. demissum* containing three *Rpi* genes (*R3*, *R4*, *R7*). A high level of resistance was observed in the 85.2% of progeny plants from the cross with resistant cultivar Kufri Jyoti, while only 36.4% of progeny were resistant in a cross with the susceptible one. A few F1 genotypes with the *RB* transgene were highly resistance to late blight, while others were completely susceptible, despite having the *RB* transgene. The authors explain that by the effects of diversity in genetic background of parental cultivars including the genes involved in signal transduction cascade and encoding pathogenesis-related proteins (Shandil et al. 2017).

Gene editing techniques are an alternative approach to introducing *Rpi* genes into potato cultivar by conventional methods or by genetic engineering. Gene editing can be used to repair non-functional alleles of *Rpi* genes. In the study by Van Doorn (2020), a non-functional allele of the *Rpi-chc1* was used from two, susceptible to late blight, cultivars Colomba and Altus. A chimeric receptor was created with exchanges in the LRR domain between the susceptible allele of the *Rpi-chc1* gene and the functional *Rpi-chc1.1* and *Rpi-chc1.2* alleles from *S. chacoense*. This resulted in the restoration of the recognition of *P. infestans* effectors *Avr-chc1.1* and *Avr-chc1.2*, which was associated with resistance to late blight (Van Doorn 2020).

Currently, transgenic potato is not available on the European market. In 2003, the transgenic potato cultivar Fortuna carrying *Rpi-blb1* and *Rpi-blb2* from *S. bulbocastanum*, which was produced by a German company BASF, was not approved for introduction in Europe (Storck et al. 2012). The American company Simplot developed Innate technology, which is used to improve known potato cultivars through genetic engineering. The second-generation Innate transgenic potato lines containing *Rpi-vnt1.1* are resistant to *P. infestans* (Richael 2021).

In conclusion, searching for new *Rpi* genes among potato wild relatives and then applying these genes in potato cultivars represents an alternative to the use of fungicides for late blight control. Due to rapid evolution of new virulent isolates of *P. infestans*, potato breeding for durable late blight resistance is challenging. The use of *Rpi* genes recognizing conservative, essential effectors of *P. infestans* and the construction of *Rpi* gene pyramids may help to achieve durable, broad-spectrum late blight resistance, which could be accelerated through genetic engineering.

### *Author contribution statement*

PP performed the literature analysis and wrote the manuscript. JŚ and ZY reviewed and corrected the manuscript. All authors contributed to the conception and design of the review.

## Data Availability

Not applicable.
